# Perception of emotional facial expressions in aggression and psychopathy

**DOI:** 10.1017/S0033291724001417

**Published:** 2024-09

**Authors:** Timo Stein, Nina Gehrer, Aiste Jusyte, Jonathan Scheeff, Michael Schönenberg

**Affiliations:** 1 Department of Psychology, University of Amsterdam, The Netherlands; 2 Department of Psychiatry and Psychotherapy, Tübingen Center for Mental Health (TüCMH), University of Tübingen, Germany; 3 Department of Clinical Psychology and Psychotherapy, University of Tübingen, Germany

**Keywords:** aggression, emotion recognition, hostile attribution bias, psychopathy, social cognition

## Abstract

**Background:**

Altered affective state recognition is assumed to be a root cause of aggressive behavior, a hallmark of psychopathologies such as psychopathy and antisocial personality disorder. However, the two most influential models make markedly different predictions regarding the underlying mechanism. According to the integrated emotion system theory (IES), aggression reflects impaired processing of social distress cues such as fearful faces. In contrast, the hostile attribution bias (HAB) model explains aggression with a bias to interpret ambiguous expressions as angry.

**Methods:**

In a set of four experiments, we measured processing of fearful and angry facial expressions (compared to neutral and other expressions) in a sample of 65 male imprisoned violent offenders rated using the Hare Psychopathy Checklist-Revised (PCL-R, Hare, R. D. (1991). *The psychopathy checklist–revised*. Toronto, ON: Multi-Health Systems) and in 60 age-matched control participants.

**Results:**

There was no evidence for a fear deficit in violent offenders or for an association of psychopathy or aggression with impaired processing of fearful faces. Similarly, there was no evidence for a perceptual bias for angry faces linked to psychopathy or aggression. However, using highly ambiguous stimuli and requiring explicit labeling of emotions, violent offenders showed a categorization bias for anger and this anger bias correlated with self-reported trait aggression (but not with psychopathy).

**Conclusions:**

These results add to a growing literature casting doubt on the notion that fear processing is impaired in aggressive individuals and in psychopathy and provide support for the idea that aggression is related to a hostile attribution bias that emerges from later cognitive, post-perceptual processing stages.

Aggressive behavior is a hallmark of several psychopathologies such as psychopathy and antisocial personality disorder. Two influential models link abnormal aggressive behavior to altered processing of social information. However, the two models differ fundamentally with regard to the proposed alterations in social information processing underlying aggression. In the integrated emotion system theory (IES), successful inhibition of aggressive impulses during development and socialization relies on accurate perception of social distress cues (Blair, 2005). In support of the IES theory, deficient perception of distress cues, e.g. of fearful or angry facial expressions, has been found in psychopathy and in antisocial populations (Brook, Brieman, & Kosson, 2013; Jusyte & Schönenberg, 2017; Marsh & Blair, 2008; Montagne et al., 2005; Schönenberg, Louis, Mayer, & Jusyte, 2013; Wilson, Juodis, & Porter, 2011; but see Dawel, O'Kearney, McKone, & Palermo, 2012). This is in marked contrast with other results showing that aggressive individuals, rather than being impaired in processing distress cues, have a heightened sensitivity for angry expressions (Mellentin, Dervisevic, Stenager, Pilegaard, & Kirk, 2015; Schönenberg & Jusyte, 2014; Wilkowski & Robinson, 2012). Such findings have been interpreted as reflecting a hostile attribution bias (HAB), where aggression results from a bias to interpret ambiguous expressions as angry, resulting in the tendency to ascribe hostile intent to others (Crick & Dodge, 1994; Dodge, 2006).

Although both models have garnered substantial empirical support, they make opposing predictions regarding the processing of social distress cues in aggressive individuals, with potentially far-reaching implications for the development of interventions and treatment (Penton-Voak et al., 2013; Schönenberg et al., 2014; but see Rosell & Siever, 2015 for a more contemporary view of mechanisms underlying aggression and violence). While the IES predicts impaired processing of fearful and perhaps angry expressions in aggressive individuals, accounts based on the HAB predict increased sensitivity for angry expressions. One possible reason for these conflicting predictions is that the two models may account for distinct stages in processing social distress cues. Indeed, the IES has been proposed to account for impaired processing at perceptual stages of stimulus encoding (Dadds, el Masry, Wimalaweera, & Guastella, 2008; Sylvers, Brennan, & Lilienfeld, 2011), whereas the HAB may reflect an interpretational bias at subsequent cognitive, post-perceptual processing stages. However, not all evidence is consistent with this levels-of-processing account. For example, deficient processing of fearful faces in aggressive individuals has sometimes been linked to impairments at later processing stages related to explicit identification, with initial fear detection and bottom-up attentional orienting to fearful faces being unimpaired (Jusyte, Stein, & Schönenberg, 2019; Stein, Jusyte, Gehrer, Scheeff, & Schönenberg, 2022). Another possibility is that the two models apply to distinct subgroups of aggressive individuals, with the IES providing a better account for instrumental (as opposed to reactive) aggression that is particularly characteristic for individuals with psychopathy (Blair, 2001; Glenn & Raine, 2009). Finally, although results from many individual studies with small sample sizes provide support for the two models, more recent work with larger sample sizes as well as meta-analyses have been calling the robustness and replicability of these findings into question (Faith, Miller, & Kosson, 2022; Hoppenbrouwers, Bulten, & Brazil, 2016).

In a set of four experiments, we measured early, perceptual, and later, post-perceptual levels of processing of fearful and angry facial expressions (compared to neutral and other expressions) in a sample of 65 male imprisoned violent offenders rated using the Hare Psychopathy Checklist-Revised (PCL-R, Hare, 1991; Hollerbach, Mokros, Nitschke, & Habermeyer, 2018) and in 60 age-matched control participants. This allowed comparing offenders and controls, as well as testing for an association between psychopathy and self-reported aggression (assessed using the German version of the Buss-Perry Aggression Questionnaire [BPAQ], Buss and Perry, 1992; Herzberg, 2003) with altered emotion processing in offenders. To test for the fear deficit predicted by the IES theory, we ran two visual search tasks in which participants searched for a target face with a different identity in an array of seven emotionally neutral, identical distractor faces (Jusyte et al., 2019). The target face could show different emotional expressions, and we expected faster visual search for targets with fearful compared to neutral expressions. In the first search task, participants indicated the target's gender, a feature for which emotion was irrelevant, so that a search advantage for fearful faces would reflect bottom-up attentional orienting (Lucas & Vuilleumier, 2008). In the second search task, to test for a fear deficit at later processing stages, participants explicitly categorized the target's emotional expression. To test for an HAB, in a separate ‘ambivalence’ experiment we presented blends of angry, fearful, and happy faces and asked participants to identify their emotions (Jusyte & Schönenberg, 2017; Schönenberg & Jusyte, 2014), expecting that aggression would be related to an increased tendency to categorize ambiguous faces as angry. Finally, to test for biases in perceptual sensitivity for both angry and fearful expressions we used a standard morphing task in which neutral faces were slowly morphed into emotional expressions and participants indicated when they first perceived any emotion in those morphs (Schönenberg et al., 2013).

## Method

### Participants, clinical, and control measures

Offenders with a primary conviction for violent offenses were recruited from cooperating German correctional facilities. We also recruited age-matched control participants. For detailed demographic and clinical sample descriptions see the Supplement and online Supplementary Table S1. Offenders and controls did not differ significantly with regard to age or cognitive abilities as assessed by the 18-item short version of the Wiener Matrizen Test (Formann, Waldherr, & Piswanger, 2011). Offenders scored significantly higher than controls on self-reported aggression (all subscales and total score of the BPAQ). On the PCL-R, offenders had a mean score of 19.4 (s.d. 8.4), with 21 offenders scoring 25 or higher, thus qualifying as psychopathic people (this subgroup had a mean PCL-R score of 28.7, s.d. 3.3).

### Visual search tasks

**Figure 1.**
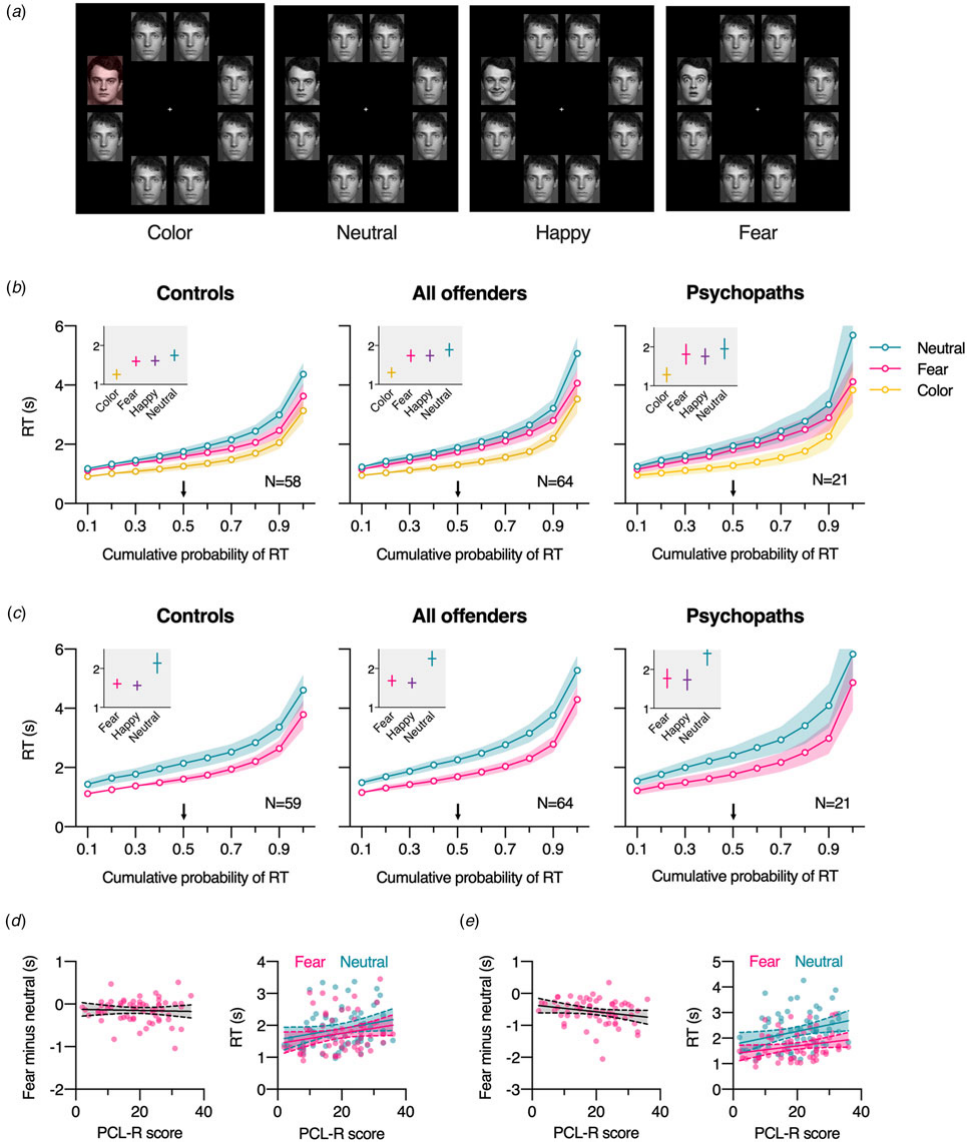
Visual search tasks. (a) Example search displays. In task 1 (gender task), participants categorized the gender of an identity singleton presented in one of eight locations of an array with seven identical, emotionally neutral distractor faces. In the two emotion conditions, the target additionally had a happy or fearful expression. In the color condition, the emotionally neutral target was additionally tinted in red. In task 2 (emotion task), participants categorized the emotion of an identity singleton presented in one of eight locations of an array with seven identical, emotionally neutral distractor faces. The target face had either a neutral, happy, or fearful expression. (b) Results from task 1 (gender task): Decile plots show RTs for the color, neutral, and fear condition, separately for control participants, all offenders, and for the subgroup of offenders diagnosed with psychopathy (PCL-R score >24). Here and in (c) the happy condition was dropped from the decile plots because of substantial overlap with the fear condition. Shaded error bars represent 95% CIs. Gray insets show median RTs with their associated 95% CIs for all four conditions. (c) Results from task 2 (emotion task). Note that there was no color condition in task 2. (d) Correlation between psychopathy as measured with the PCL-R and the task 1 fear effect (fear minus neutral, left panel) and overall median RTs in the fear and neutral condition from task 1 (right panel). (e) Correlation between PCL-R scores and the task 2 fear effect (fear minus neutral, left panel) and overall median RTs in the fear and neutral condition from task 2 (right panel). In (d) and (e) every circle represents a participant, the solid line the best-fitting linear regression line and the dashed lines the associated 95% confidence bands.

For details on the experimental procedure and stimuli for all experiments, see Supplemental Methods. In both visual search task 1 (gender task) and visual search task 2 (emotion task) participants were presented with search arrays consisting of eight faces (Jusyte et al., 2019): seven identical distractors with neutral expression and a target singleton with a different identity. Participants were instructed to press the spacebar as quickly as possible as soon as they identified the gender (task 1) or the emotional expression (task 2) of the identity singleton. After pressing the spacebar, they were asked to indicate the gender (task 1) or the expression (task 2) of the target face by pressing, without speed pressure, a labeled arrow key on the keyboard. In task 1 (gender), the target face differed from the distractors by being a different identity but also having a neutral expression (neutral condition), by being a different identity and having a happy or fearful expression (happy and fear condition), or by being a different identity and being tinted in red (color condition; see Fig. 1a). In task 2 (emotion), the search displays were identical, except that we did not include the color condition.

### Ambivalence task

Stimuli were morphs of angry, happy, and fearful expressions neutral and emotional expressions (happy, angry, fearful, sad) to create three continuous dimensions (angry/happy, angry/fearful, and fearful/happy), from which we used five distinct intensity levels, yielding

low ambiguity pairs (containing 90% and 10% of each emotion), mid ambiguity pairs (70%/30%), and high ambiguity pairs (50%/50%). Face stimuli were presented for 500 ms and participants identified the subjectively predominant expression via a corresponding button (Jusyte & Schönenberg, 2017; Schönenberg & Jusyte, 2014).

### Morphing task

Stimuli were created by morphing neutral and emotional expressions (happy, angry, fearful, sad), resulting in sequences of 51 frames, presented for 250 ms each, from completely neutral to the full-blown respective emotion. Participants pressed the space bar as soon as they recognized any emotional expression, and we calculated the mean required morphing grade for every emotion (Schönenberg et al., 2013).

### Data preprocessing, dependent variables, and statistics

For the visual search tasks, we analyzed median response times (RTs) for correct responses. For illustration purposes and to ensure that we did not miss out on differential effects in other parts of the RT distribution, we also calculated and plotted response latencies for all deciles (Fig. 1b and 1c; for analyses of response accuracies and auxiliary analyses, see Supplemental Results). For the ambivalence task, we calculated the proportion of angry and fear judgments for the three different emotion pairs. For the morphing task, we calculated the average required morphing grade for each emotion. For all tasks, participants were excluded from analyses when their grand means (across all experimental conditions) were more than 3 *SD*s above or below the group mean (across offenders and controls). For all experiments, we report both standard frequentist statistics and Bayes factors (BFs) calculated in JASP (JASP-Team, 2020) with default prior scales (Cauchy distribution, scale 0.707). When frequentist statistics indicate a significant effect, the corresponding BF quantifies the evidence for the alternative hypothesis (BF_10_); when the effect is not significant, the reported BF quantifies the evidence for the null hypothesis (BF_01_). When the assumption of sphericity was violated, we used Greenhouse–Geisser correction and report corrected degrees of freedom.

### Planned analyses

To test for differences between offenders and controls, we first analyzed the dependent variables from each task with mixed ANOVAs with a repeated-measures factor experimental condition and the between-subject factor group (offenders, controls). Next, another mixed ANOVA restricted to the sample of offenders tested for differences between individuals diagnosed with psychopathy (PCL-R > 24) and without psychopathy (PCL-R < 25). In these ANOVAs we entered all dependent variables, for example all stimulus conditions from the visual search tasks and all ambiguity and emotion conditions from the ambivalence and morphing tasks. Subsequent analyses treated psychopathy and aggression as continuous variables and correlated PCL-R and BPAQ scores with the key dependent variables from each task. The key dependent variables were those for which we expected associations with psychopathy and/or aggression. Specifically, for the visual search tasks, we ran correlations with the fear effect (difference between RTs to fearful and neutral faces) and with overall RTs to fearful faces (as well as with RTs to neutral faces as a non-emotional control). For the ambivalence task, we analyzed responses to highly ambiguous (50%/50%) faces in the fear-happy and angry-happy condition, and for the morphing task we correlated morph grades for fearful and angry faces. For additional correlation analyses including PCL-R and BPAQ sub-scores, see the Supplement (online Supplementary Tables S2 and S3).

## Results

### Visual search task 1 (gender task)

As can be seen in Fig. 1b, across the whole RT distribution the pattern across conditions was similar for offenders and controls. A mixed ANOVA on the median RTs revealed only a significant main effect of condition (*F*(2.47, 295.92) = 180.89, *p* < 0.001, *η_p_*^2^ = 0.60, BF_10_ = 1.46 × 10^68^), but no significant effect of group (*F*(1, 120) = 1.66, *p* = 0.20, *η_p_*^2^ = 0.01, BF_01_ = 1.47), and moderate evidence against an interaction between condition and group (*F*(2.47, 295.92) = 1.84, *p* = 0.15, *η_p_*^2^ = 0.02, BF_01_ = 4.97). Responses were significantly faster in the color condition (*M* = 1.28 s) than in all other conditions (*M*s = 1.67–1.82 s, Holm–Sidak's multiple comparison test, all *p*s < 0.001, all Cohen's *d*s > 1.44), and significantly slower in the neutral condition than in the happy and fear condition (*p*s < 0.001, Cohen's *d*s > 0.53), while there was no significant difference between the happy and fear condition (*p* = 0.74, Cohen's *d* = 0.03).

#### Psychopathy

A mixed ANOVA comparing the effect of condition between offenders diagnosed with psychopathy (*N* = 21) and without psychopathy (*N* = 43) revealed no significant effect of group on RTs (*F*(1, 62) = 0.13, *p* = 0.72, *η_p_*^2^ < 0.01, BF_01_ = 1.83), and, importantly, moderate evidence against a significant interaction effect (*F*(2.49, 154.07) = 1.33, *p* = 0.27, *η_p_*^2^ = 0.02, BF_01_ = 5.21; Figure 1b). Next, we correlated the fear effect with the PCL-R, such that a positive correlation would reflect a smaller effect of fear, relative to neutral, in offenders scoring higher on psychopathic traits. This correlation was not significant (*r*(62) = −0.04, *p* = 0.74, BF_01_ = 6.06; Figure 1d, left panel). The correlation between overall RTs to fearful faces with the PCL-R scores was positive but not significant (*r*(62) = 0.23, *p* = 0.069, BF_01_ = 1.28); a similar positive (non-significant) association was found when correlating RTs to neutral faces with PCL-R scores (*r*(62) = 0.23, *p* = 0.064, BF_01_ = 1.20; Figure 1d, right panel), thus providing no evidence for a fear-specific processing impairment in psychopathy.

#### Aggression

There were no significant correlations between BPAQ scores and the fear effect (*r*(120) < 0.01, *p* = 0.99, BF_01_ = 8.83) or overall RTs to fearful faces (*r*(120) = 0.09, *p* = 0.33, BF_01_ = 5.49).

### Visual search task 2 (emotion task)

Similar to the gender task, also in the emotion task the pattern of RT distributions across conditions was similar for offenders and controls (Fig. 1c). A mixed ANOVA on the median RTs revealed only a significant main effect of condition (*F*(1.23, 149.21) = 112.22, *p* < 0.001, *η_p_*^2^ = 0.48, BF_10_ = 1.54 × 10^32^), but no significant effect of group (*F*(1, 121) = 0.75, *p* = 0.39, *η_p_*^2^ < 0.01, BF_01_ = 2.56). Overall, responses were significantly slower in the neutral condition (*M* = 2.20 s) than in the happy and fear condition (Holm-Sidak's multiple comparison test, both *p*s < 0.001, both Cohen's *d*s > 1.11), while the happy and fear condition did not differ significantly (*M* = 1.60 s and *M* = 1.65 s, respectively, *p* = 0.24, Cohen's *d* = −0.11). There was strong evidence against an interaction between condition and group (*F*(1.23, 149.21) = 0.19, *p* = 0.71, *η_p_*^2^ < 0.01, BF_01_ = 16.02).

#### Psychopathy

A mixed ANOVA comparing the effect of condition between offenders diagnosed with psychopathy (*N* = 21) and without psychopathy (*N* = 43) revealed no significant effect of group on RTs (*F*(1, 62) = 1.27, *p* = 0.26, *η_p_*^2^ = 0.02, BF_01_ = 1.49), and, importantly, moderate evidence against a group-by-condition effect on RTs (*F*(1.45, 89.98) = 0.74, *p* = 0.44, *η_p_*^2^ = 0.01, BF_01_ = 5.66; Figure 1c). As for task 1, we correlated the fear effect (difference between RTs to fearful and neutral faces) with the PCL-R, and again found no significant correlation, with a trend in the opposite direction (*r*(62) = −0.23, *p* = 0.070, BF_01_ = 1.29; Figure 1e, left panel). Although the correlation between overall RTs to fearful faces with the PCL-R scores was positive, it was not significant (*r*(62) = 0.24, *p* = 0.061, BF_01_ = 1.15), and a similar positive association was found when correlating RTs to neutral faces with PCL-R scores (*r*(62) = 0.29, *p* = 0.019, BF_10_ = 2.26; Figure 1e, right panel). Thus, as for gender-search task 1, these results provide no evidence for a fear-specific deficit associated with psychopathy.

#### Aggression

There were no significant correlations between BPAQ scores and the fear effect (*r*(121) = −0.12, *p* = 0.19, BF_01_ = 3.75) or overall RTs to fearful faces (*r*(120) = 0.01, *p* = 0.90, BF_01_ = 8.79).

### Ambivalence task

**Figure 2.**
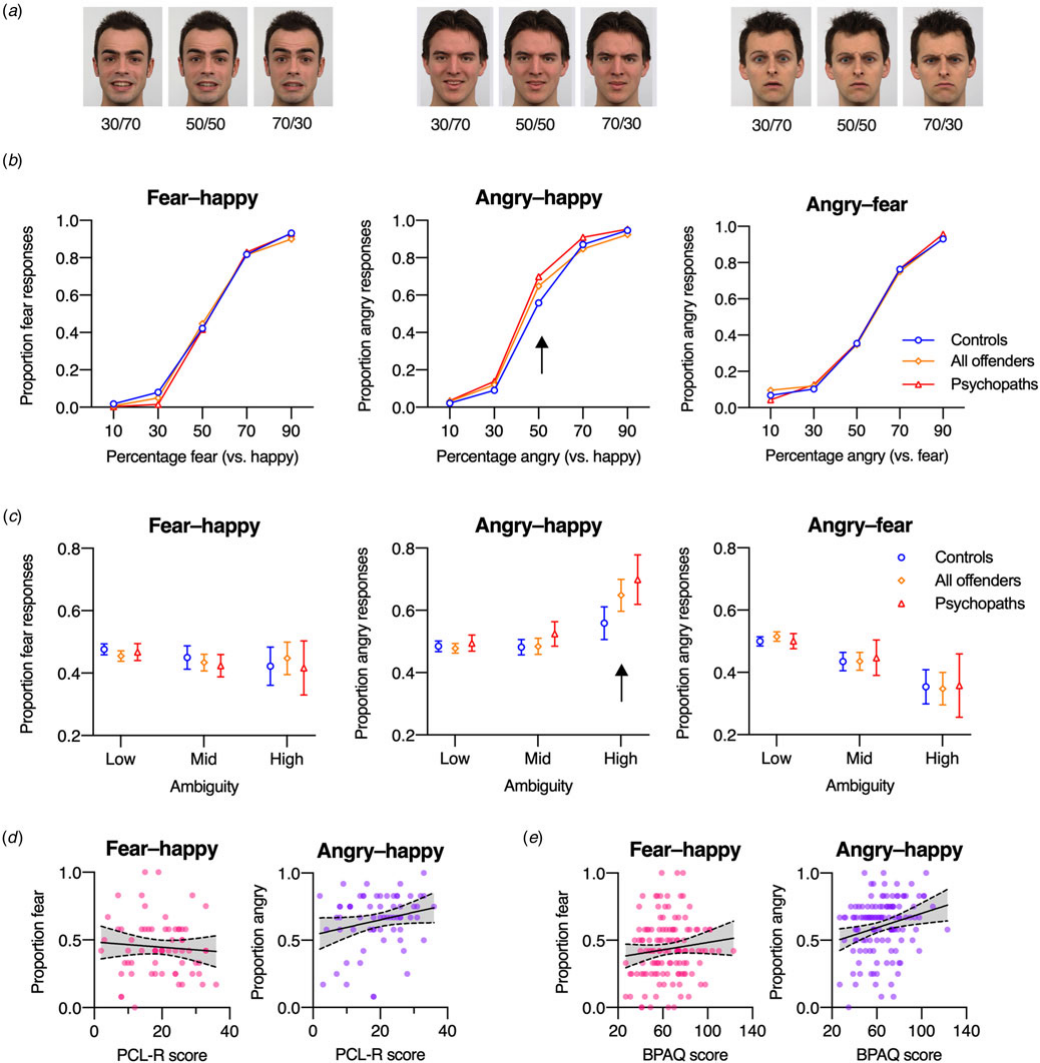
Ambivalence task. (a) Example stimuli, showing 30%/70%, 50%/50%, and 70%/30% blends for fear and happy (left), angry and happy (middle), and angry and fear (right). Participants indicated the emotion in these blends. (b). Proportion of fear/angry responses for fear-happy, angry-happy, and angry-fear blends, shown separately for controls, offenders, and for the subgroup of offenders diagnosed with psychopathy (PCL-R score > 24). (c) Proportion of fear/angry responses averaged across low ambiguity pairs (containing 90% and 10% of each emotion), mid ambiguity pairs (70%/30%), and high ambiguity pairs (50%/50%). Error bars represent 95% CIs, and the arrow highlights the key difference between controls and offenders for high-ambiguity angry-happy pairs. (d) Correlation between PCL-R scores (from offenders) and the proportion of fear/angry responses for high-ambiguity (50%/50%) fear-happy (left panel) and angry-happy (right panel) pairs. (e) Correlation between aggression as measured with the BPAQ (from all participants) and the proportion of fear/angry responses for high-ambiguity (50%/50%) fear-happy (left panel) and angry-happy (right panel) pairs. In (d) and (e), every circle represents a participant, the solid line the best-fitting linear regression line and the dashed lines the associated 95% confidence bands.

**Figure 3.**
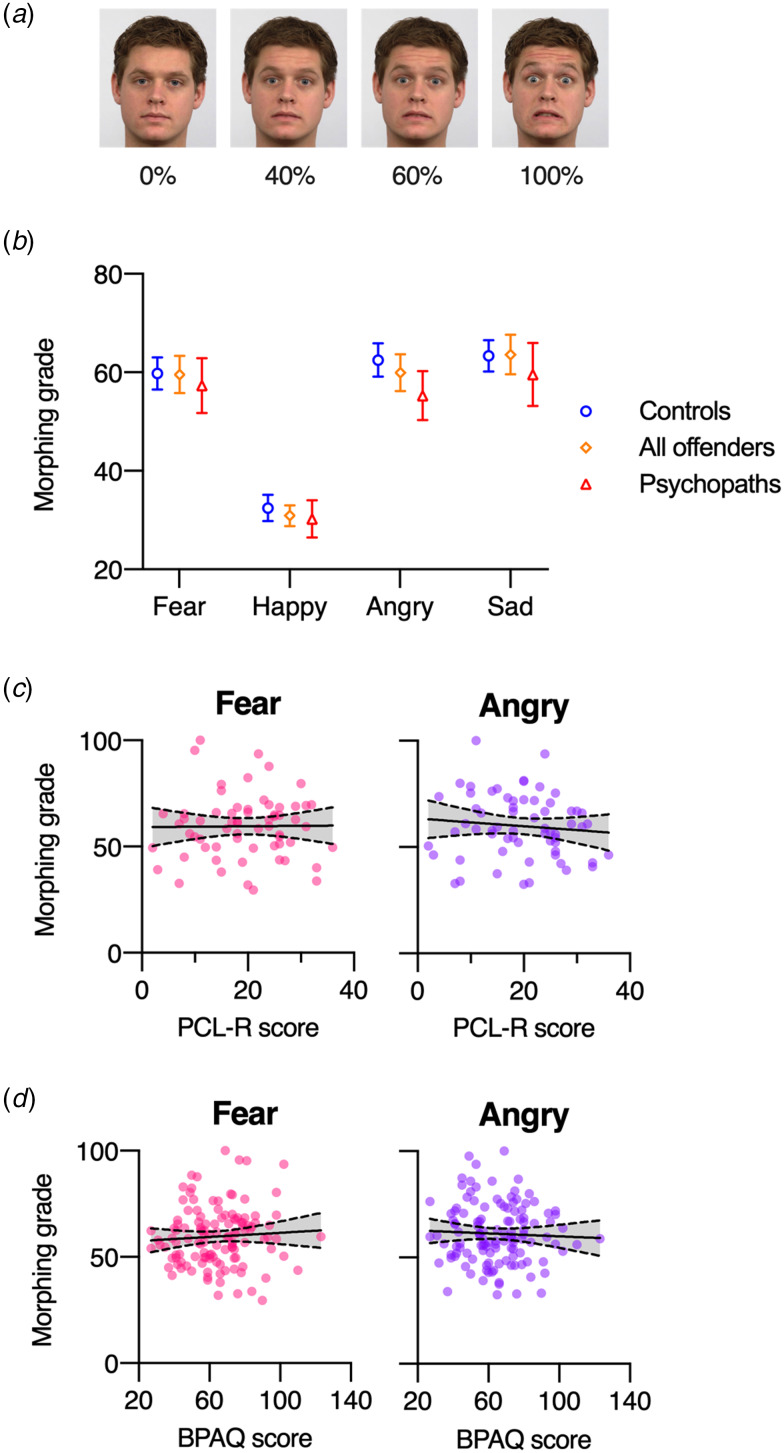
Morphing task. (a) Example morph stimuli, from all neutral (0% fear) to 100% fear. (b) Mean required morphing grade for correctly indicating an emotion, shown separately for morphs between neutral and fear, happy, angry, and sad, and for controls, offenders, and for the subgroup of offenders diagnosed with psychopathy (PCL-R score >24). Error bars represent 95% *CIs*. (c) Correlation between PCL-R scores (from offenders) and the required morphing grade for fear-morphs (left panel) and angry-morphs (right panel). (d) Correlation between BPAQ scores (from all participants) and the required morphing grade for fear-morphs (left panel) and angry-morphs (right panel). In (c) and (d), every circle represents a participant, the solid line the best-fitting linear regression line and the dashed lines the associated 95% confidence bands.

Figure 2b shows participants’ emotion judgments for the three emotion pairs (fear-happy, angry-happy, angry-fear) as a function of the percentage of each emotion contained in the face stimuli. Judgments of control participants, all offenders, and offenders diagnosed with psychopathy were almost identical for fear-happy and angry-fear pairs, but appeared to differ for angry-happy pairs, in particular for the high ambiguity condition consisting of 50% angry and 50% happy (Fig. 3).

To quantify this impression, for Fig. 2c we averaged responses to low ambiguity pairs (containing 90% and 10% of each emotion), mid ambiguity pairs (70%/30%), and high ambiguity pairs (50%/50%). This increased statistical power for analyzing the proportion of fear/angry responses in a mixed ANOVA with the factors emotion pair (fear-happy, angry-happy, angry-fear), ambiguity (low, mid, high), and group (control, offenders). While the effect of group was not significant, there were significant main effects of emotion pair and ambiguity, as well as an interaction between emotion pair and ambiguity, and an interaction between ambiguity and group (*F*(1.28, 157.48) = 3.67, *p* = 0.047, *η_p_*^2^ = 0.03, but BF_10_ = 0.25), reflecting a stronger effect of ambiguity on responses by offenders than controls, and a trend towards a significant three-way interaction (*F*(2.69, 330.41) = 2.37, *p* = 0.078, *η_p_*^2^ = 0.19, but BF_10_ = 0.11). Follow-up ANOVAs conducted separately for the three emotion-pair conditions revealed significant differences in the effect of ambiguity between offenders and controls only in the angry-happy condition (*F*(1.29, 158.61) = 6.82, *p* = 0.006, *η_p_*^2^ = 0.05, BF_10_ = 21.03), but not in the fear-happy (*F*(1.36, 167.61) = 1.44, *p* = 0.24, *η_p_*^2^ = 0.01, BF_01_ = 5.48) or angry-fear (*F*(1.46, 178.95) = 0.29, *p* = 0.68, *η_p_*^2^ < 0.01, BF_01_ = 14.14) condition. In the angry-happy condition, offenders judged high-ambiguity (50%/50%) faces significantly more often as ‘angry’ than controls (*t*(123) = 2.43, *p* = 0.016, Cohen's *d* = 0.44, BF_10_ = 2.70), while there were no significant differences between offenders and controls in the low- and mid-ambiguity conditions (both *t*(123) < 0.57, *p* > 0.56, Cohen's *d* < 0.11, BF_01_ > 4.50).

#### Psychopathy

A mixed ANOVA testing the effects of emotion pair and ambiguity between offenders diagnosed with psychopathy (*N* = 21) and without psychopathy (*N* = 44) revealed no significant interactions with participant group, with strong evidence against a three-way interaction (*F*(2.74, 85.31) = 1.11, *p* = 0.34, *η_p_*^2^ = 0.02, BF_10_ = 12.81). To test for an association of psychopathy with fear- and angry judgments of highly ambiguous stimuli, we correlated PLC-R scores with judgments of highly ambiguous (50%/50%) faces in the fear-happy and angry-happy condition. For fear judgments of fear-happy pairs with high ambiguity, there was no significant correlation (*r*(63) = −0.08, *p* = 0.54, BF_01_ = 5.37; Fig. 2d, left panel). For angry judgments of angry-happy pairs with high ambiguity, there was a trend for an increased (non-significant) tendency to judge ambiguous faces as ‘angry’ with higher PCL-R scores (*r*(63) = 0.23, *p* = 0.066, but BF_10_ = 0.81; Fig. 2d, right panel).

#### Aggression

Analogous correlation analyses revealed no significant correlation between BPAQ scores and fear judgments of fear-happy pairs with high ambiguity (*r*(123) = 0.12, *p* = 0.20, BF_01_ = 3.75; Fig. 2e, left panel) but a moderate correlation between BPAQ scores and angry judgments of angry-happy pairs with high ambiguity (*r*(123) = 0.24, *p* = 0.007, BF_10_ = 4.28; Fig. 2e, right panel). However, when restricting this analysis to the sample of offenders, the correlation between BPAQ scores and angry judgments of angry-happy pairs with high ambiguity was no longer significant *r*(63) = 0.19, *p* = 0.13, BF_01_ = 2.03; see online Supplementary Table S3).

### Morphing task

A mixed ANOVA with the within-subjects factor condition (fear, anger, happy, sad) and the between-subjects factor group (control, offenders) only yielded a significant main effect of condition (*F*(2.63, 318.60) = 456.37, *p* < 0.001, *η_p_*^2^ = 0.79, BF_10_ = 6.26 × 10^119^), reflecting a lower required morphing grade for happy (*M* = 31.7%) than for the other emotions (*M*s = 59.7–63.5%). There was no significant effect of group (*F*(1, 121) = 0.25, *p* = 0.62, *η_p_*^2^ < 0.01, BF_01_ = 3.40) and no significant interaction (*F*(2.63, 318.60) = 0.84, *p* = 0.46, *η_p_*^2^ < 0.01, BF_01_ = 17.96).

#### Psychopathy

A mixed ANOVA comparing the four emotions between offenders diagnosed with psychopathy (PCL-R > 24, *N* = 20) and without psychopathy (PLC-R < 25, *N* = 43) revealed no significant effect of group (*F*(1, 61) = 1.70, *p* = 0.20, *η_p_*^2^ = 0.03, BF_01_ = 1.43) or interaction (*F*(3, 183) = 1.55, *p* = 0.20, *η_p_*^2^ = 0.03, BF_01_ = 3.25). Also for accuracies there were no significant group or interaction effects (*F*s < 1, *p*s > 0.39, *η_p_*^2^ < 0.02, BF_01_ > 7.16). Additional correlation analyses revealed no significant correlations between PCL-R scores and morph grade for fear (*r*(61) = 0.01, *p* = 0.94, BF_01_ = 6.34) or anger (*r*(61) = −0.108, *p* = 0.43, BF_01_ = 4.69).

#### Aggression

Similarly, there were no significant correlations between BPAQ scores and morph grade for fear (*r*(121) = 0.07, *p* = 0.48, BF_01_ = 6.89) or anger (*r*(121) = −0.05, *p* = 0.62, BF_01_ = 7.83).

## Discussion

Across four experiments, our results provide no evidence for a fear deficit in violent offenders or for an association of psychopathy or aggression with impaired processing of fearful faces. These results add to a growing literature casting doubt on the idea of impaired fear processing related to aggression and psychopathy (Deming et al., 2022; Faith et al., 2022; Hoppenbrouwers et al., 2016; Stein et al., 2022). Neither did we find evidence for a perceptual bias for angry faces linked to psychopathy or aggression. However, when explicit labeling of the expression of highly ambiguous stimuli was required, we found a categorization bias for anger in violent offenders and a correlation with self-reported trait aggression (but not with psychopathy) for the whole tested sample (controls and offenders). Together, these findings support the notion that aggression is related to a hostile attribution bias that emerges from later post-perceptual ‘cognitive’ processing stages involved in explicit stimulus categorization and labeling.

There are several possible explanations for why the present study did not reveal some of the previously reported alterations in emotion processing associated with psychopathy and aggression. For example, the present explicit visual search task required mental categorization and labeling of the emotion, but in contrast to our previous study (Jusyte et al., 2019) we did not map different emotion categories on separate buttons requiring speeded responses, thus ruling out possible effects related to motor preparation and execution. We also improved the morphing task by including more trials than previous studies (Schönenberg et al., 2013, 2014), thus increasing power and reducing possible effects of differential training and familiarity with computer-based tasks between clinical and control samples.

Moreover, our study is unique in matching samples on fluid intelligence (measured with the Wiener Matrizen Test, Formann et al., 2011), while previous studies, if at all, only measured crystalline intelligence and did not take general mental ability into account. Indeed, a recent study found that when general mental ability was controlled for, psychopathy was inherently independent of deficits in the ability to perceive emotional expressions, both generally and for specific emotions. Based on their results, the authors conclude that psychopathy is genuinely associated with deficits in general mental ability and that this deficit, in turn, accounts for the observed impairments in emotion perception (Olderbak, Mokros, Nitschke, Habermeyer, & Wilhelm, 2018). Taken together, methodological issues and weaknesses including the use of heterogeneous emotion perception tasks with low power, reliance on small samples as well as insufficient control for third variable explanations might have led to the inconsistent findings reported in the field.

With regard to theoretical models that propose a link between abnormal aggressive behavior, psychopathy, and altered processing of social information, the present findings provide no empirical support that emotion perception deficits were strongest for fear, sadness, and happiness, thereby challenging central assumptions of the IES, according to which deficits should primarily concern these care-based emotions (Blair, 2001, 2005). The IES perspective posits that psychopathic individuals have difficulty inhibiting violence and aggression due to impaired recognition of these emotions, particularly of fearful expressions. The current findings, in concert with a recent study that used a signal detection theory approach (Faith et al., 2022), however, suggest that the deficits in violence inhibition seen in psychopathy reflect a mechanism other than impairments in fear recognition.

In contrast, the cognitive bias for anger associated with aggression (i.e. hostile attribution bias) appears to be independent of general mental ability and may thus reflect a genuine emotion-specific alteration in cognitive processing. We found a significantly stronger bias to categorize ambiguous angry–happy morphs as angry in offenders than in controls. Previous work suggests that this bias is particularly related to reactive rather than instrumental aggression (Philipp-Wiegmann, Rösler, Retz-Junginger, & Retz, 2017), which might explain the correlation with aggressive behavior but not psychopathy in the current study. It should be noted, however, that this correlation was significant only when considering the whole tested sample (controls and offenders), but not when restricting the analysis to offenders only, as for all correlations with psychopathy scores. This observation is consistent with a recent review that reported small to medium associations between the hostile attribution bias and aggression in adults across different samples (Klein Tuente, Bogaerts, & Veling, 2019). The authors found no evidence for a stronger association between the hostile attribution bias and aggression in groups displaying higher levels of aggression (e.g. forensic patients, offenders) than in students or general populations. In general, more aggressive individuals attribute more hostile intentions to people in ambiguous and/or clearly hostile social situations. The association between HAB and aggression therefore seems to capture a general mechanism underlying aggression rather than a pathological relation.

Further research is however required to examine whether the hostile attribution bias in face perception represents a general precursor (e.g. in aggression-prone youths), how it relates to subtypes of aggressive behavior, whether it may be modified by conventional therapeutic strategies or even represent a possible target for new computer-based training approaches. Indeed, there is first evidence that hostile attributions may be reduced with relatively simple means, such as implicit bias modification (Penton-Voak et al., 2013; Stoddard et al., 2016). Such experimental manipulations may help understand the dynamics of biased decision-making processes and simultaneously inform effective intervention strategies.

As with other empirical studies, the present study has some limitations. First, we included only a moderately sized sample of male inmates, a relatively rare sample of violent offenders spanning a single developmental period, thus compromising generalizability to female offenders and/or nonincarcerated individuals high in psychopathy or aggression. Second, we only studied psychopathy in a clinical sense, restricting diagnosis of psychopathic traits to offenders, so that we could not examine associations with psychopathy in the absence of violent offending. However, development of and research using the PCL-R has been based on samples of offenders and forensic psychiatric patients, so that this conflation is common in research on psychopathy. Future work could include other groups such as community samples with psychopathic traits. Furthermore, we mostly focused on attentional aspects of emotion processes and exclusively on facial processing, and the stimulus material exclusively consisted of male faces. Future studies should therefore employ a more complete and ecologically valid stimulus set and tasks that go beyond attentional processes. For example, we did not include expressions of all basic emotions in all experiments and only used static images, so that we may have missed potential processing alterations for specific dynamic facial expressions (Decety, Skelly, Yoder, & Kiehl, 2014).

Relatedly, and more fundamentally, some recent evidence challenges the widespread assumption of a universal, uniform, and stable production of six facial expressions of basic emotion and argues for a new, more dynamic view of the function, morphology, and signal value of facial behavior (Durán & Fernández-Dols, 2021). Facial movements commonly thought to signal particular emotions regardless of context, person, and culture are not universally diagnostic of emotional states, and thus future research should employ multimodal approaches by utilizing data from multiple sources, such as facial expressions, posture and gait, tone of voice, or gaze to allow a more comprehensive and accurate assessment of human emotional states (Barrett, Adolphs, Marsella, Martinez, & Pollak, 2019). Because of these limitations, our findings do not refute the extensive literature linking emotional processing mechanisms with aggressive behaviors.

In conclusion, across four experiments we found no evidence for perceptual deficits for emotion (including fear) in psychopathy, but a cognitive bias for anger linked to aggression. These results challenge the view that psychopathy arises from altered emotion processing and support the idea that a hostile attribution bias may underlie aggressive behavior.

## Supporting information

Stein et al. supplementary material Stein et al. supplementary material

## Supplementary material

The supplementary material for this article can be found at https://doi.org/10.1017/S0033291724001417.
