# Supplementary material for: Perception of emotional facial expressions in aggression and psychopathy
Source: Psychol Med. 2024 Sep 9;54(12):3294–302. doi: 10.1017/S0033291724001417 (PMC11496222; doi:10.1017/S0033291724001417)
Supplement: Stein et al. supplementary material [file S0033291724001417sup001.docx]

**Perception of emotional facial expressions in aggression and psychopathy**

***Supplemental Methods and Results***

Timo Stein^1^, Nina Gehrer^2,3^, Aiste Jusyte^2^, Jonathan Scheeff^2,3^ and Michael Schönenberg^2,3^

^1^Department of Psychology, University of Amsterdam, The Netherlands

^2^Department of Psychiatry and Psychotherapy, University Hospital of Tübingen, Germany

^3^Department of Clinical Psychology and Psychotherapy, University of Tübingen, Germany

**Author Note**

Correspondence concerning this article should be addressed to Timo Stein, Department of Psychology, University of Amsterdam, Nieuwe Achtergracht 129B, 1001 NK Amsterdam, The Netherlands. Email: t.stein@uva.nl

**Supplemental Methods**

**Participants**

Offenders were recruited from cooperating German correctional facilities (Justizvollzugsanstalten Heimsheim, Rottenburg, Hohenasperg) through advertisement via pamphlets and blackboards within the facilities. The advertisement specified that we sought inmates aged 18–65 years with a primary conviction for violent offenses and sufficient knowledge of the German language. Interested individuals were contacted by the facility’s psychological service to schedule the assessments. All assessments were carried out by trained psychologists from our research group (unrelated to the correctional facility in any way) in designated rooms of the facility. All assessors were bound to confidentiality regarding any information about the mental health status disclosed during assessments or the behavioral test results. Exclusion criteria were primary conviction for drug-related crime, psychotic spectrum, or bipolar disorders (as assessed by clinical interview). Control participants were recruited through advertisements in newspapers and the university’s mailing list (inclusion criteria: 18–65 years, no self-reported convictions, no ASPD, no history of bipolar or psychosis-spectrum disorder). Individual participant data were coded via pseudonyms and were at no point accessible to the correctional facility’s staff members. The study was approved by the local ethics committee and was conducted in accordance with the Declaration of Helsinki. Participants received monetary compensation.

**Testing procedure and apparatus**

Prior to the experimental tasks, all participants were notified about the purposes of the study and signed a written informed consent form. All participants completed the German version of *The Buss-Perry Aggression Questionnaire (BPAQ)*. The BPAQ consists of 29 self-administered items rated on a 5-point Likert scale and was developed in order to evaluate four dimensions of aggression: (a) physical aggression, (b) verbal aggression, (c) anger, and, (d) hostility. The score for each scale is the sum or the ratings for its items. The two items (7 and 18) worded in the direction opposite to aggression are reverse-scored. The total score for aggression is the sum of these scale scores. Internal consistency is high (sum score: α = 0,88; verbal aggression scale: α = 0,65; physical aggression 2: α = 0,86; anger: α = 0,76; hostility: α = 0,75; Herzberg, 2003).

For the imprisoned offenders, trained independent psychologists of the correctional facilities had assigned the scores of the *Hare Psychopathy Checklist* – *Revised (PCL-R)* as part of a standard diagnostic procedure. The PCL-R is a 20-item diagnostic tool that was developed in the 1990s and is frequently used in forensic settings to rate a person’s psychopathic and/or antisocial tendencies (Hare, 1993). The response format is a 3-point scale, where 0 = item does not apply; 1 = item applies somewhat; 2 = applies definitely. Assessors obtain PCL-R total scores by summing all 20 individual PCL-R items, but can also obtain two factor scores and four facet scores by summing subsets of items. Factor 1 captures the interpersonal (Facet 1) and affective (Facet 2) aspects of psychopaths (e.g., superficial charm, lack of remorse, and lack of guilt). Factor 2 assesses behavior associated with a socially deviant lifestyle (Facet 3) and antisocial behavior (Facet 4) (e.g., impulsivity and juvenile delinquency. In the German adaption, a score of 25 or greater corresponds to a diagnosis of psychopathy. The PCL-R is not used in isolation to perform a diagnosis, but in combination with a semi-structured interview and a review of the client’s file records and history. Interrater-reliability for the German version (Hollerbach et al., 2018) is good (ICC = 0,77), and internal consistency high (sum score: α = 0,85; factor 1: α = 0,82; factor 2: α = 0,84).

Following the diagnostic assessments, the participants were introduced to the experimental tasks. For the controls, all assessments were carried out in unitary settings in laboratory rooms. The offender group data were gathered in designated rooms within the facility; none of the security or psychological service staff members were present in the room during testing. The experiments were run on 14.1’’ HP laptops at a viewing distance of approximately 40 cm. For the search tasks, stimulus presentation and data collection were controlled by MATLAB (The MathWorks, Natick, MA) using the Psychtoolbox functions (Brainard, 1997). For the ambivalence and the morphing task, Presentation® software (Version 17.0, Neurobehavioral Systems, Inc., Berkeley, CA) was used.

**Visual search tasks**

***Stimuli.*** We selected 32 photographs (16 female identities) with neutral, happy, and fearful expressions from the FACES Database (Ebner et al., 2010) . The photographs were cropped to a rectangle and converted to grayscale. For the color condition, faces with neutral expression were tinted red by changing the color balance (adding 24 pixel values to the red channel and subtracting 12 pixel values from the green and blue channel, respectively). Additional eight faces (four female) were selected for practice trials.

***Procedure.*** Every trial began with a 1.6-s presentation of a central white fixation cross, which was followed by a visual search array shown until response. Task 1 consisted of 128 trials in which each combination of four target conditions (color, neutral, happy, fear), two target genders and 16 target identities per gender occurred once. Trial order was randomized. The target could appear in any of the eight positions within the search array, with the constraint that each target position occurred twice per target condition. The specific distractor identities were randomized, with the constraints that for every condition in half of the trials the distractors had the same gender as the target and in the other half had the same gender, and that a given distractor identity occurred only once per condition. The gender of the distractors was therefore not predictive of the target gender. There was one obligatory break after 64 trials. All participants first completed a brief practice block of eight trials. Task 2 always followed task 1 to avoid carry-over effects from the explicit emotion-categorization task 2 to the incidental-processing task 1. The design of task 2 was identical to that of task 1, except that the color condition was dropped, resulting in a total of 96 trials (with an obligatory break after 48 trials, and eight practice trials before starting the experiment proper).

***Analysis and statistics.*** The main dependent variable was response time (RT), which we calculated as the median response latency for correct responses (excluding trials with latencies shorter than 0.1 s and longer than 10 s, 0.2% of all trials) for every participant and condition. We also calculated mean accuracy rates for every participant and condition in order to rule out potential speed-accuracy trade-offs between groups or condition (see below). Participants were excluded from analyses when their median RT or accuracy across all experimental conditions were more than 3 *SD*s above or below the group mean (across offenders and controls). For both visual search tasks, this resulted in the exclusion of one offender and two control participants.

**Ambivalence task**

***Stimuli.*** Color photographs of three male models depicting emotional expressions (angry, happy and fearful) were selected from the Radboud Faces (Langner et al., 2010). All images were cropped and subsequently blended (FantaMorph software, Abrosoft, Beijing, China) with each other to create three continuous dimensions (angry/happy, angry/fearful, and fearful/happy). For each dimension, we created five distinct intensity levels containing different proportions of each blended emotion (e.g., a 90% angry and 10% happy, a 70% angry and 30% happy, a 50% angry and 50% happy (maximal ambiguity), a 30% angry and 70% happy, and a 10% angry and 90% happy blend for the ‘angry/happy’ dimension). Therefore, the stimulus material for the experiment consisted of 45 distinct images (3 model identities × 3 emotional expressions × 5 intensity levels). For the practice trials, 15 additional stimuli (3 emotional expressions × 5 intensity levels) of one male model (code 15) were created in the same manner and were not used in the proper experiment.

***Procedure.*** All images (scaled to a height of 500 pixels) were centered on a gray background. Participants completed 15 practice trials and began the experimental task with a total of 180 trials (3 models × 3 emotions × 5 intensity levels × 4 repetitions), which were pseudo-randomized across emotions and intensity ratios (i.e., no more than two identical consecutive trials in a row). Each trial started with a fixation cross presented for 1000 ms that was then replaced by a face stimulus (500 ms). Participants were asked to identify the subjectively predominant emotional expression depicted by the stimulus (happy, angry, fearful) on a forced-choice task (open-ended response time) by logging their response via a corresponding button. The intertrial interval was randomly selected to be 1000 or 1500 ms long (Jusyte & Schönenberg, 2017; Schönenberg & Jusyte, 2014).

***Analysis and statistics.*** The dependent variable was the mean proportion of angry and fear judgments for the three different emotion pairs. For our main analysis, we increased statistical power by averaging responses to low ambiguity pairs (containing 90% and 10% of each emotion), mid ambiguity pairs (70%/30%), and high ambiguity pairs (50%/50%), respectively.

**Morphing task**

***Stimuli.*** Color photographs of ten models (five female) depicting neutral and emotional (happy, angry, fearful, sad) expressions were selected from the Radboud Faces Database (Langner et al., 2010). Images were cropped and the neutral and emotional expressions of every model identity were blended (FantaMorph software, Abrosoft, Beijing, China), resulting in six distinct neutral-emotional sequences for each model. For every sequence, a set of 51 frames with intensity levels ranging from completely neutral to the full-blown respective emotion were extracted (2% increment steps). The mouth and teeth area of the ambiguous expressions were adjusted for color and luminance using Adobe Photoshop CS4 to make for a seamless transitions between stimuli. Three photographs of an additional model identity were used to create blends between neutral and disgusted as well as between neutral and surprised expressions for practice trials using the same procedure.

***Procedure.*** Participants were introduced to the animated morph task, which depicted a video sequence of a neutral face slowly developing into a surprised or disgusted expression. Stimuli were presented at the center of the computer screen against a uniform grey background and scaled to the height of 500 pixels. The trial structure of the morph task was as follows: 51 morphed images depicting increasing intensity levels of an emotional expression, starting with a neutral expression and ending with a full-blown emotional expression, were each presented for 250 ms with no intertrial intervals. This created the impression of a gradually developing video sequence of a neutral face developing into an emotional expression. Participants were instructed to press the space bar as soon as they were able to correctly recognize the depicted expression. The sequence was then immediately stopped, the face disappeared, and the selected emotion category were recorded. The experiment consisted of a total of 40 trials (10 model identities x 4 emotional expressions) presented in randomized order.

***Analysis and statistics.*** The main dependent variable was required morph grade, as reported in the main manuscript. We also analyzed accuracy in reporting the respective emotion to rule out potential speed-accuracy trade-offs between groups or conditions (see below). Participants were excluded from analyses when their required morphing grade or accuracy across all experimental conditions were more than 3 *SD*s above or below the group mean (across offenders and controls). This resulted in the exclusion of two offenders.

Table S1

*Demographic Diagnostic Sample Description*

| Variable | | Offender  (N = 65) | Controls  (N = 60) | Statistics | Cohen’s d (95% CI) | | Bayes factor | |
| --- | --- | --- | --- | --- | --- | --- | --- | --- |
| Age | | 40.86 (11.73) | 43.40 (11.42) | t(123) = −1.23 | −0.22 (−0.57, 0.13) | | BF_01_ = 2.66 | |
| WMT sum score | | 7.40 (3.95) | 7.05 (3.40) | t(123) = 0.53 | 0.01 (−0.26, 0.45) | | BF_01_ = 4.61 | |
| BPAQ | |  |  |  |  |  |  |  |
|  | Physical aggression | 22.09 (9.03) | 14.97 (5.85) | t(123) = 5.19*** | 0.93 (0.56, 1.30) | | BF_10_ = 1.64×10^4^ | |
|  | Verbal aggression | 14.68 (3.65) | 13.00 (3.21) | t(123) = 2.72** | 0.49 (0.13, 0.84) | | BF_10_ = 5.18 | |
|  | Anger | 14.95 (5.13) | 10.77 (3.94) | t(123) = 5.09*** | 0.91 (0.54, 1.28) | | BF_10_ = 1.09×10^4^ | |
|  | Hostility | 21.48 (6.62) | 16.87 (6.19) | t(123) = 4.01*** | 0.72 (0.36, 1.08) | | BF_10_ = 210.74 | |
|  | Total score | 73.20 (17.86) | 55.60 (15.60) | t(123) = 5.58*** | 1.05 (0.67, 1.42) | | BF_10_ = 2.54×10^5^ | |

*Note.* The data refer to mean sum scores and standard deviations for each measure (in parentheses). WMT = Wiener Matrizen Test. BPAQ = Buss-Perry Aggression Questionnaire . ***p* < .01. ****p* < .001.

**Supplemental Results**

In order to rule out differential speed/accuracy trade-offs, we also analyzed accuracy rates for the two visual search tasks and for the morphing task.

**Visual search task 1 (gender task) – accuracy**

Overall accuracy was high (controls: *M* = 97.8% correct, offenders: *M* = 97.5% correct). A mixed ANOVA with the within-subjects factor condition (color, neutral, happy, fear) and the between-subjects factor group (control, offenders) only yielded a significant main effect of condition (*F*(3, 360) = 6.21, *p* < .001, η*_p_*^2^ = .05, BF_10_ = 28.26), reflecting higher accuracy in the color condition (*M* = 98.4% correct) than in the neutral, happy, and fear conditions (*M*s = 97.0–97.7% correct). There was no significant effect of group (*F*(1, 120) = 0.37, *p* = .54, η*_p_*^2^ < .01, BF_01_ = 4.77) and no significant interaction (*F*(3, 360) = 0.74, *p* = .53, η*_p_*^2^ < .01, BF_01_ = 19.63). Thus, response accuracy in both groups was boosted by the increased physical saliency of color targets.

An additional mixed ANOVA comparing the effect of condition between offenders diagnosed with psychopathy (PCL-R > 24, *N* = 21) and without psychopathy (PLC-R < 25, *N* = 43) revealed no significant effect of group on accuracies (*F*(1, 62) = 0.57, *p* = .46, η*_p_*^2^ < .01, BF_01_ = 1.28×10^8^), and moderate evidence against a significant interaction effect (*F*(3, 186) = 0.80, *p* = .49, η*_p_*^2^ = .01, BF_01_ = 7.79).

**Visual search task 2 (emotion task) – accuracy**

Overall accuracy was high (controls: *M* = 97.4% correct, offenders: *M* = 97.0% correct). A mixed ANOVA with the within-subjects factor condition (neutral, happy, fear) and the between-subjects factor group (control, offenders) only yielded a non-significant trend for a main effect of condition (*F*(2, 242) = 2.82, *p* = .058, η*_p_*^2^ = .02, BF_01_ = 2.35), reflecting somewhat higher accuracy in the neutral condition (*M* = 97.9% correct) than in the happy (*M* = 97.0% correct) and fear (*M* = 96.7% correct) condition. There was no significant effect of group (*F*(1, 121) = 0.39, *p* = .53, η*_p_*^2^ < .01, BF_01_ = 5.51) and no significant interaction (*F*(2, 242) = 0.39, *p* = .68, η*_p_*^2^ < .01, BF_01_ = 12.88).

An additional mixed ANOVA comparing the effect of condition between offenders diagnosed with psychopathy (PCL-R > 24, *N* = 21) and without psychopathy (PLC-R < 25, *N* = 43) revealed no significant effect of group on accuracies (*F*(1, 62) = 2.07, *p* = .155, η*_p_*^2^ = .03, but BF_01_ = 0.16) and moderate evidence against a group-by-condition effect on accuracies (*F*(2, 124) = 0.44, *p* = .65, η*_p_*^2^ < .01, BF_01_ = 6.79).

**Morphing task – accuracy**

A mixed ANOVA with the within-subjects factor emotion (fear, anger, sad, happy) and the between-subjects factor group (control, offenders) revealed a significant main effect of emotion *F*(3, 363) = 62.86, *p* < .001, η*_p_*^2^ = .34, BF_10_ = 8.59×10^29^), reflecting much higher overall accuracy for happy (*M* = 99.7% correct) than for the other emotions (*M*s = 79.6–85.9% correct), but no significant main effect of group (*F*(1, 121) = 0.26, *p* = .61, η*_p_*^2^ < .01, BF_01_ = 6.26) and no significant interaction (*F*(3, 363) = 2.56, *p* = .06, η*_p_*^2^ = .02, BF_01_ = 2.02).

An additional mixed ANOVA comparing the emotion effect between offenders diagnosed with psychopathy (PCL-R > 24, *N* = 20) and without psychopathy (PLC-R < 25, *N* = 43) revealed no significant main effect of group (*F*(1, 61) = 0.11, *p* = .74, η*_p_*^2^ < .01, BF_01_ = 3.50×10^13^) and no significant interaction (*F*(2.68, 163.73) = 0.77, *p* = .50, η*_p_*^2^ = .01, BF_01_ = 9.41).

**Additional correlation analyses including PCL-R and BPAQ sub-scores**

We conducted additional correlation analyses of the key behavioral measures from the two visual search tasks (Table S2) and from the ambivalence and morphing tasks (Table S3) with the two factors and the four facets of the PCL-R, as well as with the four factors of the BPAQ. Note that for some offenders, only the total score of the PCL-R was available. We also repeated the correlations with the BPAQ for the sample of offenders only (Tables S2 and S3).

Table S2

*Additional correlation analyses for visual search tasks 1 and 2*

| Variable | | Visual search 1  Fear effect | | Visual search 1  Overall RTs | | | Visual search 2  Fear effect | | | Visual search 2  Overall RTs | | |
| --- | --- | --- | --- | --- | --- | --- | --- | --- | --- | --- | --- | --- |
| PCL-R (offender only) | |  | |  | | |  | | |  | |  |
| Factor 1 | | *r*(58) = -.08, *p* = .55 | | Fear: *r*(58) = .32, *p* = .013  Neu: *r*(58) = .34, *p* = .008 | | | *r*(58) = -.22, *p* = .095 | | | Fear: *r*(58) = .33, *p* = .011  Neu: *r*(58) = .36, *p* = .005 | | |
| Factor 2 | | *r*(58) < .01, *p* = .99 | | Fear: *r*(58) = .08, *p* = .53  Neu: *r*(58) = .08, *p* = .55 | | | *r*(58) = -.12, *p* = .38 | | | Fear: *r*(58) = .07, *p* = .58  Neu: *r*(58) = .11, *p* = .39 | | |
| Interpersonal | | *r*(58) = -.06, *p* = .65 | | Fear: *r*(58) = .19, *p* = .15  Neu: *r*(58) = .21, *p* = .12 | | | *r*(58) = -.12, *p* = .37 | | | Fear: *r*(58) = .19, *p* = .15  Neu: *r*(58) = .20, *p* = .12 | | |
| Affective | | *r*(58) = -.09, *p* = .50 | | Fear: *r*(58) = .39, *p* = .002  Neu: *r*(58) = .42, *p* < .001 | | | *r*(58) = -.29, *p* = .026 | | | Fear: *r*(58) = .41, *p* = .001  Neu: *r*(58) = .46, *p* < .001 | | |
| Lifestyle | | *r*(58) = .05, *p* = .72 | | Fear: *r*(58) = .08, *p* = .53  Neu: *r*(58) = .06, *p* = .66 | | | *r*(58) = -.08, *p* = .57 | | | Fear: *r*(58) = .10, *p* = .45  Neu: *r*(58) = .11, *p* = .39 | | |
| Antisocial | | *r*(58) = -.03, *p* = .83 | | Fear: *r*(58) = .03, *p* = .83  Neu: *r*(58) = .04, *p* = .76 | | | *r*(58) = -.12, *p* = .35 | | | Fear: *r*(58) = .01, *p* = .91  Neu: *r*(58) = .07, *p* = .59 | | |
| Total score | | *r*(62) = -.04, *p* = .74 | | Fear: *r*(62) = .23, *p* = .069  Neu: *r*(62) = .23, *p* = .064 | | | *r*(62) = -.23, *p* = .070 | | | Fear: *r*(62) = .24, *p* = .061  Neu: *r*(62) = .29, *p* = .019 | | |
| BPAQ (full sample) | |  |  | |  |  | |  |  | |  | |
|  | Physical aggression | *r*(120) = .03, *p* = .74 | | Fear: *r*(120) = .04, *p* = .66  Neu: *r*(120) = .02, *p* = .79 | | | *r*(121) = -.02, *p* = .84 | | | Fear: *r*(121) = -.06, *p* = .54  Neu: *r*(121) = -.02, *p* = .84 | | |
|  | Verbal aggression | *r*(120) = .02, *p* = .84 | | Fear: *r*(120) = -.02, *p* = .83  Neu: *r*(120) = -.03, *p* = .78 | | | *r*(121) = -.24, *p* = .007 | | | Fear: *r*(121) = -.05, *p* = .60  Neu: *r*(121) = .14, *p* = .13 | | |
|  | Anger | *r*(120) = .07, *p* = .44 | | Fear: *r*(120) = .14, *p* = .12  Neu: *r*(120) = .10, *p* = .27 | | | *r*(121) = -.09, *p* = .32 | | | Fear: *r*(121) = .05, *p* = .58  Neu: *r*(121) = .09, *p* = .32 | | |
|  | Hostility | *r*(120) = -.10, *p* = .29 | | Fear: *r*(120) = .11, *p* = .25  Neu: *r*(120) = .13, *p* = .14 | | | *r*(121) = -.12, *p* = .21 | | | Fear: *r*(121) = .09, *p* = .33  Neu: *r*(121) = .13, *p* = .16 | | |
|  | Total score | *r*(120) < .01, *p* = .99 | | Fear: *r*(120) = .09, *p* = .33  Neu: *r*(120) = .08, *p* = .37 | | | *r*(121) = -.12, *p* = .19 | | | Fear: *r*(121) = .01, *p* = .90  Neu: *r*(121) = .09, *p* = .33 | | |
| BPAQ (offender only) | |  |  | |  |  | |  |  | |  | |
|  | Physical aggression | *r*(62) = .02, *p* = .87 | | Fear: *r*(62) < .01, *p* = .99  Neu: *r*(62) < .01, *p* = .95 | | | *r*(62) = -.07, *p* = .58 | | | Fear: *r*(62) = -.09, *p* = .46  Neu: *r*(62) = -.03, *p* = .82 | | |
|  | Verbal aggression | *r*(62) = .09, *p* = .46 | | Fear: *r*(62) < .01, *p* = .96  Neu: *r*(62) = -.05, *p* = .71 | | | *r*(62) = -.27, *p* = .032 | | | Fear: *r*(62) = -.10, *p* = .45  Neu: *r*(62) = .07, *p* = .56 | | |
|  | Anger | *r*(62) = .04, *p* = .74 | | Fear: *r*(62) = .23, *p* = .07  Neu: *r*(62) = .20, *p* = .12 | | | *r*(62) = -.33, *p* = .008 | | | Fear: *r*(62) = .13, *p* = .31  Neu: *r*(62) = .27, *p* = .031 | | |
|  | Hostility | *r*(62) = -.11, *p* = .38 | | Fear: *r*(62) = .19, *p* = .13  Neu: *r*(62) = .23, *p* = .07 | | | *r*(62) = -.19, *p* = .13 | | | Fear: *r*(62) = .14, *p* = .28  Neu: *r*(62) = .20, *p* = .11 | | |
|  | Total score | *r*(62) < .01, *p* = .99 | | Fear: *r*(62) = .14, *p* = .28  Neu: *r*(62) = .13, *p* = .32 | | | *r*(62) = -.26, *p* = .037 | | | Fear: *r*(62) = .02, *p* = .87  Neu: *r*(62) = .15, *p* = .22 | | |

Table S3

*Additional correlation analyses for the ambivalence and the morphing task*

| Variable | | Ambivalence task  50% Fear, 50% Happy | | | Ambivalence task  50% Angry, 50% Happy | | | | Morphing task  Fear morph grade | | | | Morphing task  Anger morph grade | | |
| --- | --- | --- | --- | --- | --- | --- | --- | --- | --- | --- | --- | --- | --- | --- | --- |
| PCL-R (offender only) | |  | |  | | |  | | | |  | | |  | |
| Factor 1 | | *r*(59) = .05, *p* = .71 | | | *r*(59) = .22, *p* = .090 | | | | *r*(57) = .06, *p* = .68 | | | | *r*(57) = -.07, *p* = .62 | | |
| Factor 2 | | *r*(59) = -.19, *p* = .14 | | | *r*(59) = .18, *p* = .18 | | | | *r*(57) = -.09, *p* = .48 | | | | *r*(57) = -.19, *p* = .16 | | |
| Interpersonal | | *r*(59) = .14, *p* = .30 | | | *r*(59) = .16, *p* = .21 | | | | *r*(57) = -.13, *p* = .32 | | | | *r*(57) = -.24, *p* = .068 | | |
| Affective | | *r*(59) = -.07, *p* = .62 | | | *r*(59) = .23, *p* = .070 | | | | *r*(57) = .27, *p* = .042 | | | | *r*(57) = .16, *p* = .22 | | |
| Lifestyle | | *r*(59) = -.11, *p* = .39 | | | *r*(59) = .05, *p* = .71 | | | | *r*(57) = -.05, *p* = .70 | | | | *r*(57) = -.22, *p* = .096 | | |
| Antisocial | | *r*(59) = -.22, *p* = .085 | | | *r*(59) = .16, *p* = .23 | | | | *r*(57) = -.14, *p* = .28 | | | | *r*(57) = -.15, *p* = .26 | | |
| Total score | | *r*(63) = -.08, *p* = .54 | | | *r*(63) = .23, *p* = .066 | | | | *r*(61) = .01, *p* = .094 | | | | *r*(61) = -.10, *p* = .43 | | |
| BPAQ (full sample) | |  |  | | |  | |  | |  | |  | | |  |
|  | Physical aggression | *r*(123) = .06, *p* = .50 | | | *r*(123) = .21, *p* = .017 | | | | *r*(121) = .06, *p* = .53 | | | | *r*(121) = -.11, *p* = .25 | | |
|  | Verbal aggression | *r*(123) = .13, *p* = .16 | | | *r*(123) = .25, *p* = .006 | | | | *r*(121) = .06, *p* = .53 | | | | *r*(121) = .02, *p* = .87 | | |
|  | Anger | *r*(123) = .09, *p* = .33 | | | *r*(123) = .25, *p* = .005 | | | | *r*(121) = .02, *p* = .83 | | | | *r*(121) = .01, *p* = .90 | | |
|  | Hostility | *r*(123) = .12, *p* = .19 | | | *r*(123) = .10, *p* = .29 | | | | *r*(121) = .06, *p* = .48 | | | | *r*(121) = -.01, *p* = .89 | | |
|  | Total score | *r*(123) = .12, *p* = .20 | | | *r*(123) = .24, *p* = .007 | | | | *r*(121) = .07, *p* = .48 | | | | *r*(121) = -.05, *p* = .62 | | |
| BPAQ (offender only) | |  |  | | |  | |  | |  | |  | | |  |
|  | Physical aggression | *r*(63) = .08, *p* = .54 | | | *r*(63) = .17, *p* = .17 | | | | *r*(61) = -.02, *p* = .85 | | | | *r*(61) = -.18, *p* = .17 | | |
|  | Verbal aggression | *r*(63) = .16, *p* = .21 | | | *r*(63) = .21, *p* = .096 | | | | *r*(61) = .02, *p* = .85 | | | | *r*(61) = .04, *p* = .75 | | |
|  | Anger | *r*(63) = .01, *p* = .92 | | | *r*(63) = .25, *p* = .044 | | | | *r*(61) = -.09, *p* = .50 | | | | *r*(61) = -.02, *p* = .87 | | |
|  | Hostility | *r*(63) = .09, *p* = .49 | | | *r*(63) = -.03, *p* = .79 | | | | *r*(61) < .01, *p* = .96 | | | | *r*(61) = .03, *p* = .85 | | |
|  | Total score | *r*(63) = .11, *p* = .40 | | | *r*(63) = .19, *p* = .13 | | | | *r*(61) = -.04, *p* = .79 | | | | *r*(61) = -.08, *p* = .54 | | |

**Supplemental references**

Brainard, D. H. (1997). The psychophysics toolbox. *Spatial Vision*, *10*, 433–436.

Ebner, N. C., Riediger, M., & Lindenberger, U. (2010). FACES – A database of facial expressions in young, middle-aged, and older women and men: Development and validation. *Behavior Research Methods*, *42*(1), 351–362. https://doi.org/10.3758/BRM.42.1.351

Hare, R. D. (1993). Without conscience : the disturbing world of the psychopaths among us. In *Guilford Press*.

Herzberg, P. Y. (2003). Faktorstruktur, Gütekriterien und Konstruktvalidität der deutschen Übersetzung des Aggressionsfragebogens von Buss und Perry. *Zeitschrift Für Differentielle Und Diagnostische Psychologie*, *24*(4). https://doi.org/10.1024/0170-1789.24.4.311

Hollerbach, P., Mokros, A., Nitschke, J., & Habermeyer, E. (2018). Hare Psychopathy Checklist-Revised: German language adaptation and recommendations for correct use. *Forensische Psychiatrie, Psychologie, Kriminologie*, *12*(3). https://doi.org/10.1007/s11757-018-0478-z

Jusyte, A., & Schönenberg, M. (2017). Impaired social cognition in violent offenders: perceptual deficit or cognitive bias? *European Archives of Psychiatry and Clinical Neuroscience*, *267*(3), 257–266. https://doi.org/10.1007/s00406-016-0727-0

Langner, O., Dotsch, R., Bijlstra, G., Wigboldus, D. H. J., Hawk, S. T., & van Knippenberg, A. (2010). Presentation and validation of the Radboud faces database. *Cognition and Emotion*, *24*(8), 1377–1388. https://doi.org/10.1080/02699930903485076

Schönenberg, M., & Jusyte, A. (2014). Investigation of the hostile attribution bias toward ambiguous facial cues in antisocial violent offenders. *European Archives of Psychiatry and Clinical Neuroscience*, *264*(1), 61–69. https://doi.org/10.1007/s00406-013-0440-1
